# A Retrospective Analysis of the Relationship between Ethnicity, Body Mass Index, and the Diagnosis of Gestational Diabetes in Women Attending an Australian Antenatal Clinic

**DOI:** 10.1155/2015/297420

**Published:** 2015-10-01

**Authors:** Rebecca McDonald, Amalia Karahalios, Thao Le, Joanne Said

**Affiliations:** ^1^Monash Women's, Monash Health, 246 Clayton Road, Clayton, VIC 3168, Australia; ^2^Women's and Children's Health, Sunshine Hospital, Western Health, 176 Furlong Road, Saint Albans, VIC 3021, Australia; ^3^Office for Research, Western Centre for Health Research & Education, 176 Furlong Road, Saint Albans, VIC 3021, Australia; ^4^Centre for Epidemiology and Biostatistics, Melbourne School of Population and Global Health, University of Melbourne, 207 Bouverie Street, Carlton, VIC 3053, Australia; ^5^Department of Obstetrics and Gynaecology, The University of Melbourne, Parkville, VIC 3052, Australia; ^6^Maternal Fetal Medicine, Sunshine Hospital, Western Health, 176 Furlong Road, Saint Albans, VIC 3021, Australia

## Abstract

*Purpose*. To estimate the prevalence of gestational diabetes mellitus (GDM) in a multiethnic population, assess the association between country of birth (COB) and GDM, and assess whether the association varies by body mass index (BMI). *Methods*. A retrospective study of 5260 pregnant women attending Sunshine Hospital, Australia, between 1st July 2012 and 30th June 2013. We fitted logistic regression models to assess the association between COB and GDM. An interaction between BMI and COB was assessed by likelihood ratio test. *Results*. In the 4610 included in our analysis, most common were women born in Australia or New Zealand (ANZ, 1932, 41.9%) and in Southeast Asia (922, 20%). GDM was diagnosed in 606 (13.2%) women. After adjusting for confounders, women from East Asia were most likely to develop GDM (37, 24.0%) and 5-fold more likely than women from ANZ (OR = 4.77, 95% CI: 3.12, 7.31, *p* < 0.001). Women from other Asian countries had a 3-fold increased risk of GDM compared to women from ANZ. There was no evidence of an interaction by BMI (*p* = 0.24).  *Conclusions*. Women born in Asia have higher risk of GDM compared to women born in ANZ. These data provide support for including COB in GDM management policies.

## 1. Introduction

Gestational diabetes mellitus (GDM) is a condition that affects 6.5% of pregnant women in Australia [1] and is increasing in prevalence in Australia and worldwide [2, 3]. It is associated with wide reaching, sometimes long term and potentially severe, effects for both the mother and her child many of which can be ameliorated by lifestyle and pharmacological treatment of GDM [4]. These include increased perinatal mortality rates, major pregnancy, labour, and postdelivery complications, and an increased risk of obesity and metabolic syndrome in the offspring [2, 5–7]. However, there is increasing recognition that GDM may present and behave differently in women of different ethnicities and backgrounds. This makes it important to understand specific local population characteristics when designing and implementing local services. Australia's multicultural composition is frequently described as being intrinsic to Australian identity. One in four Australians were born overseas and 44% were either born overseas or had a parent who was, and this number is increasing [8, 9]. The proportion of Australians born in Europe has declined in recent years while there has been a significant increase in the populations of Australians who were born in Central, South, and Southeast Asia [9]. Western Health, in Melbourne, Victoria, Australia, services a very ethnically diverse population. In particular, the region hosts a large South Asian and Southeast Asian community who are known to be at particular risk of developing GDM [10]. Migrant women of any ethnicity are more prone to GDM in comparison to women of both their birth and host countries although reasons for this may be varied [11, 12].

Race/ethnicity and obesity are two of the strongest independent risk factors for GDM [13–17]. There is a positive association between an unhealthy weight and the development of GDM seen for all ethnicities but the strength of the association appears to be variable between ethnicities. The strongest association is found in women of South Asian and Black African ethnicities whereas the association seems to be weaker for other Asian groups [15, 16]. One study reported that the association between body mass index (BMI) and GDM appeared to plateau at a BMI around 28 kg/m^2^ in Asian groups but continued past a BMI of 35 km/m^2^ in other groups [16]. Pregnant Asian women appear to have a greater degree of insulin resistance than Caucasian women at similar BMIs and demonstrate a stronger association between prepregnancy BMI and insulin resistance. This may partly be explained by a greater central adiposity and percentage of body fat in people of Asian descent [17].

The World Health Organisation (WHO) has recognised that a healthy weight range, based on studies of risk factors and morbidities, varies with the ethnicity of the population studied [17] and may be lower (e.g., Southeast Asian populations) or higher (e.g., Pacific Islanders) than for Caucasians [18]. Therefore, cut-offs derived from European data do not provide an adequate basis for taking action on risks related to overweight and obesity. It suggests that the increased risk of health problems associated with increasing BMI must be regarded as being on a continuum with increasing BMI [17].

Local populations must be well understood in order to develop and implement policies that will provide the greatest benefit. A previous evaluation, in 1991, of GDM prevalence in a similar Australian population has found that rates may be tripled in women of Indian subcontinental and other Asian origins compared to women from Australia and New Zealand (ANZ) [19]. Australia is increasingly multiethnic and the prevalence of GDM is known to be increasing [2, 3, 19, 20] making reevaluation pertinent. This is particularly relevant given the changing immigration patterns over time. The current study aimed to determine the prevalence of GDM in a widely multiethnic population of women who presented to Western Health for pregnancy care and assess whether the association between body mass index (BMI) and GDM prevalence is modified by ethnicity.

## 2. Materials and Methods

### 2.1. Ethical Approval

This study has been approved by the Western Health Low Risk ethics panel (QA Reference Number: QA2014.111) and it conforms to the provisions of the Declaration of Helsinki (as revised in Seoul, 2008).

### 2.2. Design and Participants

All women who gave birth at Sunshine Hospital between 1st July 2012 and 30th June 2013 were included in this retrospective cohort study. Women with diabetes mellitus types one (T1DM) or two (T2DM) were excluded. We also excluded women who presented to the hospital with an already established diagnosis of GDM in the current pregnancy. Sunshine Hospital receives referrals from smaller centres of women excluded from care at these centres following their diagnosis with GDM. Excluding these women from analysis avoided overestimation of GDM as a result of these referrals. We used BOS (Birthing Outcome System), a specialised pregnancy related clinical information system, to access demographics and pregnancy outcomes data. The outcome measure was diagnosis of GDM. The maternal BMI was recorded at the first visit. Cases with missing BMI, country of birth (COB), age, or oral glucose tolerance test (OGTT) data were excluded. Also, cases with a BMI recorded as less than 16 were excluded in order to eliminate cases in which a patient's height or weight had been omitted or incorrectly entered into the height or weight fields. Cases with a BMI greater than 50 were manually reviewed to ensure their accuracy.

Our centre used the Australian Diabetes in Pregnancy Society (ADIPS) guidelines for the testing and diagnosis of GDM initially published in 1991 [20]. In accordance with this guideline, women were diagnosed with GDM if one or both of the following criteria were met during 75 g glucose bolus OGTT:

- Fasting blood glucose level ≥5.5 mmol/L.
- 2 hours after a 75 g glucose bolus, blood glucose level ≥8.0 mmol/L.

Women underwent risk based screening in early pregnancy. Remaining women and women in whom OGTT was negative on early screening underwent universal screening at 24 to 26 weeks gestation.

For each woman identified through BOS as having a diagnosis of GDM, we confirmed that the results of the OGTT were concordant with a diagnosis of GDM according to the ADIPS criteria. We then extracted further information regarding any prior OGTTs and the endocrinological management after diagnosis of GDM.

Patients were considered to have GDM regardless of the gestational age at diagnosis so long as pregestational T1DM or T2DM was excluded.

Screening and management protocols were consistent with the ADIPS guidelines and included routine testing with an OGTT for all women (without a prior glucose challenge test) at 26–28 weeks except where tested for and diagnosed earlier in pregnancy. Management involved early consultation with a credentialed diabetes educator and a dietician and self-monitoring of capillary blood glucose before and after meals. Insulin therapy was initiated if women were unable to meet ADIPS treatment targets with dietary and lifestyle modification. Adherence to these treatment guidelines was not assessed in this study.

COB, established by self-report at first visit, was used as a proxy for ethnicity. Women were then allocated to one of the following 10 ethnic subgroups: Europe and North America, the Arab States, West and Central Asia, Southeast Asia, East Asia, South Asia, Africa, Latin America, Oceania, and Australia and New Zealand (ANZ). These groups are described in Table 1.

### 2.3. Data Analysis

Parity was grouped into nulliparous (parity = 0), multiparous (parity ≥ 1 and < 5) and grand-multiparous (parity ≥ 5). BMI was grouped into underweight (<18.5 kg/m^2^), normal weight (≥18.5 and <25 kg/m^2^), overweight (≥25 and <30 kg/m^2^), obese (≥30 and <35 kg/m^2^), and morbidly obese (≥35 kg/m^2^) according to WHO groupings [14]. Maternal age was grouped in quartiles (determined by the overall maternal ages of women delivering at the hospital during this time period), with quartile 1 defined as age ≤26 years, quartile 2 as age >26 and ≤29, quartile 3 as age >29 and ≤33, and quartile 4 as >33.

We fitted a univariable logistic regression model to assess the association between COB and presence or absence of GDM. Next, we fitted a multivariable logistic regression model to assess the above-mentioned association (i.e., the association between COB and GDM) after adjusting for age, parity, and BMI. We hypothesised* a priori* that the association between COB and GDM might vary by BMI. We fitted an interaction term for COB and BMI and tested the interaction with the likelihood ratio test.

We used the likelihood ratio test to test the assumption of a (log) linear association between age and GDM. We did this by fitting a model with age grouped into quartiles and compared it to a model with age as a pseudocontinuous variable (set to the median value in each quartile). There was evidence of a nonlinear association between age and (log odds) of GDM; therefore age was included as a categorical variable.

Data were collated using Excel 2013 and were analysed using SPSS v. 20 (IBM Corp., 2011).

## 3. Results

**Figure 1:**
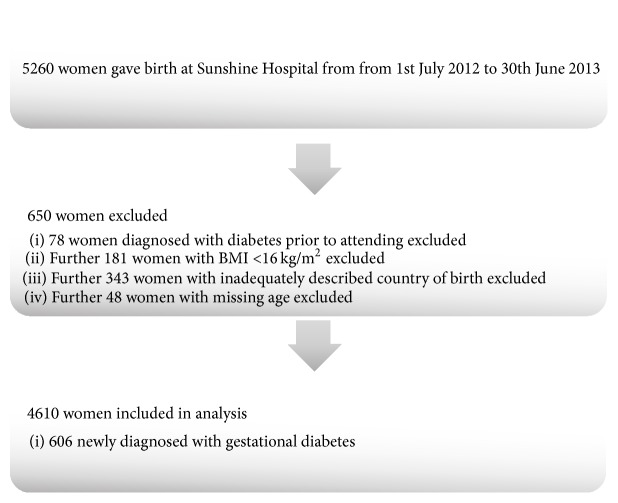
Flow chart of women who gave birth at Sunshine Hospital from 1st July 2012 to 30th June 2013 showing numbers included in and excluded from study.

Of the 5260 women who attended Sunshine Hospital for delivery between 1st July 2012 and 30th June 2013, 650 were excluded from our analysis. 78 women were identified as either having pregestational T1DM or T2DM or were referred to Sunshine Hospital for care after being identified as having GDM. An additional 181 women were excluded due to incomplete BMI entries. COB was inadequately described in a further 343 women and age was missing in a further 48 cases. Of the 4610 women included in our analyses, 606 (13.2%) women were newly diagnosed with GDM (Figure 1).

The majority of women were born in Australia or New Zealand (*n* = 1932 (41.9%)). Other major COB groups were Southeast Asia (*n* = 922, 20.0%) and South Asia (*n* = 673, 14.6%). Overall, 40.9% of women were born in an Asian country (*n* = 1887). The mean age of our antenatal population was 29.2 years (Standard Deviation (SD) = 6.1 years), median BMI was 25.0 kg/m^2^ (Interquartile range (IQR) 22.0–29.0). Also, 1998 (43.3%) were nulliparous and 2612 (56.7%) were multiparous (parity ≥ 1) (Table 2).

Table 3 presents the results from univariable and multivariable logistic regression analysis. After adjusting for age, BMI, and parity, women born in East Asia had almost 5-fold increased odds of GDM compared to women born in ANZ (OR = 4.77; 95% confidence interval (CI) = 3.12, 7.31, *p* value < 0.001). Similarly, women born in West and Central Asia, South Asia, and Southeast Asia had an approximately 3-fold increased risk of GDM (OR for West and Central Asia = 2.47, 95% CI 1.50–4.05, *p* = 0.01; OR for South Asia = 3.38, 95% CI 2.60–4.40, *p* < 0.001; OR for South East Asia = 3.03, 95% CI 2.34–3.93, *p* < 0.001). There was no evidence of an interaction between BMI and COB (*p* from likelihood ratio test = 0.24).

## 4. Discussion

This study found that women born in West and Central Asia, Southeast Asia, East Asia, or South Asia had the highest risks of GDM compared to women born in Australia or New Zealand. Ethnicity and BMI are recognised to have the strongest association with GDM.

Hedderson et al. (2012) [16] reported that increasing BMI is associated with an increased prevalence of GDM for all ethnic groups but that this effect was stronger for White, Hispanic, and African American women than for Asian women, whereas Kim et al. (2013) found the strongest association between Indian and Black African women and the weakest between other Asian groups [15]. However, Kim et al. also found substantial subgroup variability in the Asian cohort [15]. An interaction between COB and BMI in the association with GDM was not found in our data. In our study, there were very few Asian women who were obese and morbidly obese. This may have limited our ability to completely explore such an interaction. In practice though, the low prevalence of obese and morbidly obese Asian women must limit the utility of using traditional BMI cut-offs to predict GDM in Asian women. However, the findings of the present study do not support the use of racially specific BMI cut-offs in screening protocols. This is in line with the most recent WHO guidance. The WHO has recognised that BMI cut-offs for observed health risks are very specific to relatively small ethnic groups [17] and therefore suggests treating BMI associated risk as a continuum.

The reasons why migrant Asian women have a higher risk of GDM are diverse and unclear. Asian persons are at greater risk of T2DM, the aetiology of which may partially explain their predisposition for GDM [21]. Asian women display greater insulin resistance in pregnancy after age, weight gain in pregnancy, and history of diabetes are removed, and the association between prepregnancy BMI and insulin resistance is greater [22]. Renzaho et al. (2010) [12] cite a disruption of normal eating habits, dietary acculturation, lack of physical activity, and rapid weight gain after dietary restriction as possible lifestyle mediators. Bandyopadhyay et al. (2011) [3] reported that South Asian migrant women with culturally different food and exercise habits reported difficulty initiating and sustaining appropriate diet and exercise regimens.

The present study was limited by being retrospective. It is recognised that country of birth may not always reflect ethnicity. In particular, the population of women born in Australia or New Zealand may already be ethnically diverse. This may have increased the heterogeneity of our groups. We were unable to control for other factors commonly associated with the development of GDM such as diet and exercise. It has been previously suggested that diet and exercise may explain part of the association between COB and GDM (discussed above). Also, we sampled very small numbers of obese and morbidly obese women, despite a large sample size. This may have limited our ability to completely explore an interaction between BMI and COB as discussed above.

Regardless of the reasons for which ethnicity affects risk of diabetes, this study, performed in a multiethnic population, supports the need for ethnicity to be included in GDM screening as well as in management guidelines and policies. There is a need for widespread early screening in at-risk ethnic groups and for early implementation of culturally sensitive management techniques that may ameliorate the barriers identified by Bandyopadhyay et al. (2011) [3]. Future studies may investigate ethnic differences in the development of early onset GDM when all women are tested prior to 20 weeks gestation and investigate the utility of early interventions.

## Figures and Tables

**Table 1:** List of included countries by region.

Region	Country of birth			
Europe and North America	Albania	Herzegovina	Russia	Wales
	Belgium	Hungary	Scotland	Bermuda
	Bosnia	Ireland	Serbia	Canada
	Bulgaria	Italy	Slovakia	United States
	Croatia	Lithuania	Slovenia	
	England	Malta	Spain	
	Finland	Montenegro	Sweden	
	Former Yugoslav Republic of Macedonia	Norway	Switzerland	
	France	Poland	Ukraine	
	Germany	Portugal	Oman	
	Greece	Romania	United Kingdom	

Arab States	Bahrain	Jordan		United Arab Emirates
	Egypt	Kuwait	Saudi Arabia	Yemen
	Iraq	Lebanon	Syria	

West and Central Asia	Afghanistan	Iran	Pakistan	Turkmenistan
	Cyprus	Nepal	Turkey	

South East Asia	Cambodia	Laos	Thailand	Papua New Guinea
	East Timour	Malaysia	Philippines	Vietnam
	Indonesia	Myanmar	Singapore	

East Asia	China	Japan	Macau	
	Hong Kong	Korea (South)	Taiwan	

South Asia	Bangladesh	India	Sri Lanka	

Africa	Burundi	Ethiopia	Libya	Somalia
	Chad	Gambia	Mauritius	Sudan
	Comoros	Ghana	Morocco	Tanzania
	Congo	Guinea	Nigeria	Togo
	Djibouti	Kenya	Rwanda	Uganda
	Eritrea	Liberia	Sierra Leone	Zimbabwe

Latin America	Argentina	Colombia	Mexico	Uruguay
	Brazil	El Salvador	Panama	
	Chile	Guatemala	Peru	

Oceania	Cook Islands	Nauru	Samoa	Tonga
	Fiji	Niue	Tokelau	Vanuatu

ANZ	Australia	New Zealand		

**Table 2:** Demographic characteristics of women included in the study group.

Maternal characteristics	Number (%) (*N* = 4610)
Country of birth	
Australia or New Zealand	1932 (41.9)
Arab States	111 (2.4)
West and Central Asia	138 (3.0)
Southeast Asia	922 (20.0)
East Asia	154 (3.3)
South Asia	673 (14.6)
Africa	354 (7.7)
Latin America	41 (0.9)
Oceania	83 (1.8)
Europe and North America	202 (4.4)
Age (years)^†^	29.2 ± 6.1
1st quartile (≤26 years)	1360 (29.5)
2nd quartile (>26 and ≤29 years)	1009 (21.9)
3rd quartile (>29 and ≤33 years)	1186 (25.7)
4th quartile (>33 years)	1055 (22.9)
Parity	
Nulliparous (parity = 0)	1998 (43.3)
Multiparous (parity 1 to 4)	2484 (53.9)
Grand multiparity (parity ≥ 5)	128 (2.8)
Body mass index (kg/m^2^)	
Underweight (<18.5)	198 (4.3)
Normal (≥18.5 and <25)	2066 (44.8)
Overweight (≥25 and <30)	1231 (26.7)
Obese (≥30 and <35)	556 (12.1)
Morbidly obese (≥35)	559 (12.1)

^†^Mean and standard deviation.

**Table 3:** Results of univariable and multivariable logistic regression for presence or absence of gestational diabetes mellitus (GDM) in 4610 women at Sunshine Hospital, Australia.

Characteristics	GDM		Univariable analysis		Multivariable^†^ analysis	
	GDM (*n*)	No GDM (*n*)	Odds ratio (95% CI)	*p* value	Odds ratio (95% CI)	*p* value
Country of birth						
Australia or New Zealand	175	1757	1.00		1.00	
Arab States	12	99	1.21 (0.65, 2.24)	0.55	1.33 (0.71, 2.50)	0.37
West and Central Asia	22	116	1.89 (1.17, 3.06)	0.01	2.47 (1.50, 4.05)	<0.001
Southeast Asia	161	761	2.11 (1.68, 2.66)	<0.001	3.03 (2.34, 3.93)	<0.001
East Asia	37	117	3.16 (2.11, 4.71)	<0.001	4.77 (3.12, 7.31)	<0.001
South Asia	136	537	2.59 (2.03, 3.30)	<0.001	3.38 (2.60, 4.40)	<0.001
Africa	34	320	1.12 (0.77, 1.63)	0.56	1.24 (0.83, 1.84)	0.29
Latin America	4	37	0.73 (0.42, 1.29)	0.28	0.90 (0.51, 1.60)	0.72
Oceania	11	72	1.06 (0.37, 3.00)	0.91	0.94 (0.33, 2.70)	0.91
Europe and North America	14	188	1.52 (0.79, 2.91)	0.21	1.12 (0.57, 2.19)	0.74
Age (per year)						
25th centile (<26 years)	144	1216	1.00		1.00	
50th centile (≥26 and <29 years)	121	888	1.15 (0.89, 1.49)	0.28	1.02 (0.78, 1.33)	0.88
75th centile (≥29 and <33 years)	146	1040	1.19 (0.93, 1.51)	0.17	1.05 (0.82, 1.35)	0.71
≥75th centile (≥33 years)	195	860	1.91 (1.52, 2.42)	<0.001	1.70 (1.34, 2.17)	<0.001
Body mass index (kg/m^2^)						
Underweight (<18.5)	22	176	0.92 (0.64, 1.32)	0.64	0.96 (0.60, 1.54)	0.87
Normal (≥18.5 and <25)	221	1845	1.00		1.00	
Overweight (≥25 and <30)	184	1047	1.47 (1.20, 1.81)	<0.001	1.72 (1.38, 2.14)	<0.001
Obese (≥30 and <35)	84	472	1.50 (1.15, 1.95)	0.003	2.14 (1.60, 2.86)	<0.001
Morbidly obese (≥35)	95	464	1.70 (1.32, 2.20)	<0.001	3.16 (2.34, 4.27)	<0.001
Parity						
Nulliparous (parity = 0)	259	1739	1.00		1.00	
Multiparous (parity 1 to 4)	321	2163	1.01 (0.85, 1.19)	0.96	1.00 (0.83, 1.19)	0.98
Grand multiparity (parity ≥5)	26	102	1.73 (1.12, 2.66)	0.01	1.80 (1.13, 2.86)	0.01
Total	606	4004				

^†^The multivariable analyses are adjusted for the other remaining maternal characteristics listed in the table. Nagelkerke *R* ^2^ = 0.08.

95% CI: 95% confidence interval.
